# Insulin Sensitivity from Preschool to School Age in Patients with Severe Obesity

**DOI:** 10.1371/journal.pone.0068628

**Published:** 2013-07-31

**Authors:** Melania Manco, Maria Rita Spreghini, Rosa Luciano, Cecilia Pensini, Rita Wietrzycowska Sforza, Carmela Rustico, Marco Cappa, Giuseppe Stefano Morino

**Affiliations:** 1 Scientific Directorate, Research Unit for Multifactorial Disease, Bambino Gesù Children Hospital, Rome, Italy; 2 Unit for Clinical Nutrition, Bambino Gesù Children Hospital, Rome, Italy; 3 Unit of Endocrinology and Diabetology, Bambino Gesù Children Hospital, Rome, Italy; 4 Department of Laboratory Medicine, Bambino Gesù Children Hospital, Rome, Italy; University of Texas Health Science Center at San Antonio, United States of America

## Abstract

**Background:**

Insulin sensitivity decreases at puberty transition, but little information has been provided on its earlier time-course. Aim of the present study was to describe the time-course of insulin sensitivity in severely obese children at the transition from preschool to school age.

**Research design and methods:**

Retrospective study of a cohort of 47 severely obese [Body Mass Index (BMI) ≥99° percentile] preschoolers evaluated twice, once between 2 and 6 years of age, and once before age 8. Glucose tolerance, Whole Body Insulin Sensitivity Index (WBISI), Insulinogenic Index (IGI); β-cell demand index (BCDI) and Insulin Secretion-Sensitivity Index 2 (ISSI-2) were longitudinally estimated during the oral glucose tolerance test.

**Results:**

After a median follow-up of 2.23 (1–4.52) y, obese patients showed significant decrease in WBISI (p<0.0001), and increase in fasting (p = 0.005) and 2 h glucose (2HG, p = 0.001). One child in preschool age and 4 school age children presented with 2HG between 7.8–11.1 mmol/l. Best predictors of WBISI, 2HG and BCDI in the school age were changes in BMI z-score (R^2^ = 0.309; p = 0.002; β = −0.556), ISSI-2 (R^2^ = 0.465; p<0.0001; β = −0.682), and BMI z-score (R^2^ = 0.246; p = 0.008; 0.496), respectively.

**Conclusions:**

In morbidly obese children, insulin sensitivity seems to decline even before pubertal transition, but changes in total adiposity can only partially explain this variation.

## Introduction

The unabating rise in the prevalence of childhood obesity has been accompanied by the emergence of impaired glucose metabolism (IGM) in young people [1]–[2]. In obese individuals, IGM results from enhanced insulin resistance and impaired ability to compensate for augmented β-cell demand [3]–[4].

Insulin resistance occurs at pubertal transition during a time of profound change in body composition and hormone levels [5]. Enhanced insulin resistance has been related to changes in body fatness [6], sex steroids [7] and growth hormone/IGF-1 levels [8]. Studies have clearly demonstrated that while pre-pubertal and post-pubertal individuals are equally sensitive to insulin, pubertal children become more insulin resistant probably to favor the acceleration in body growth and the body's transition to adult appearance [5]–[11].

In contrast to the consistent literature on the pathogenesis of IGM in prepubertal (age 6 years onward), peripubertal and teenage obese individuals [1], [3]–[4], [6]–[11], little is known about the underlying mechanisms implicated in the development of these disorders in children before the age of 6 y. Large cohort studies of healthy children, i.e. the Early Bird Diabetes study [12] and the Bogalusa Heart study [13]), have provided data on the time-course of insulin resistance from prepuberty to puberty, but were limited to fasting estimation of insulin resistance by using the homeostasis model assessment of insulin resistance (HOMA-IR), suggesting that the decline of insulin sensitivity begins years before onset of puberty. Development of insulin resistance at such an early age may lead to early development of hypertension, dyslipidemia and fatty liver disease via mechanisms which have been widely investigated in school-age children and adolescents [14].

To the best of our knowledge, there has been no longitudinal study on the interplay between insulin resistance and the capability of the β-cell to eventually adapt to enhanced insulin demand in obese preschoolers, both estimated using indexes derived from the oral glucose tolerance test (OGTT).

Our study aimed at retrospectively describing the time-course of parameters of glucose metabolism (i.e., glucose tolerance, insulin sensitivity, β-cell function and glucose disposition index) in a sample (N = 47) of severely obese children followed from preschool (2–6 y old) to school age (7–8 y old).

## Subjects and Methods

### Participants

At the Clinical Nutrition Unit of the Bambino Gesù Children's Hospital, patients referred for obesity [Body Mass index (BMI) ≥95^th^ percentile for age and sex] by general pediatricians undergo a standard clinical evaluation protocol which includes recording of anthropometrics, blood pressure, lipid profile, liver function tests, uric acid, 5 time-point OGTT as previously described [15]–[16]. Medical records for 47 severely obese Caucasian children (BMI≥99th percentile), aged 2–6 y, were retrospectively analyzed. The patients were selected from amongst those consecutively referred to the Unit from January 2006 to December 2011 to exclude known genetic, syndromic or endocrine disorders. Inclusion criteria were age, two complete data sets (the first evaluation between 2 and 6 y, and the second before age 8 y), no initial pubertal development (Tanner stage I), no previous treatment for obesity, no systemic or endocrine disease, no medication.

The BMI z-score [17] and percentiles of waist circumference [18] were both calculated using US reference values. Systolic (SBP) and diastolic blood pressure (DBP) were measured three times while the subjects were seated, and the measurements averaged for the analysis. Puberty development was clinically assessed on the basis of secondary sex characteristics. The configuration of the breasts and the quantity and pattern of pubic hair determine the ratings of girls. Genital development and the quantity and pattern of pubic hair determine the ratings of boys. Tanner stages for pubic hair, breast configuration, and genital status were used as reference [19]. None of the subjects had started puberty.

The study protocol has been approved by the Ethical Committee of the Bambino Gesù Children's Hospital. Written and oral information was given to parents/carers, before written full informed consent was obtained in order to use patient's data for research purposes. The study protocol conformed to the guidelines of the European Convention of Human Rights and Biomedicine for Research in Children and to those of the Ethics Committee of the “Bambino Gesù” Hospital. All measures have been taken to ensure the confidentiality of families and children participating. In particular, Directive 95/46/EC of the European Parliament and of the Council of 24 October 1995 on the protection of personal data will be have been complied with for data storage and handling in order to ensure patient data protection and confidentiality.

### Oral glucose tolerance test

Glucose tolerance was classified according to the criteria of the American Diabetes Association classification [20]. A standard OGTT (1.75 g/kg body weight up to a maximum of 75 g) was performed with flavored glucose (Glucosio Sclavo Diagnostics, 75 g/150 ml) after 8 hour overnight fast. After local application of an anesthetic cream (EMLA Cream), one anti-cubital i.v. catheter was inserted for blood sampling and was maintained patent by a normal saline drip during the test. Blood samples were obtained every 30 minutes for 120 minutes for the measurement of serum glucose and insulin.

The Homeostasis Model Assessments of fasting Insulin Resistance [21] and the Whole Body Insulin Sensitivity Index were computed [22]. The area under the curve (AUC) was estimated using the trapezoidal rule and with glucose, insulin and time expressed in mmol/l, pmol/l and minutes, respectively. Insulin secretion was estimated by means of the insulinogenic index [23] and the ratio 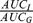. The glucose disposition index was calculated as Insulin Secretion-Sensitivity Index-2, ISSI-2 [24]. The ratio of IGI and WBISI was computed to derive the β-cell demand index (BCDI) according to Weiss *et al* [25].

### Analytical methods

Blood was kept in ice and assays were performed within one hour in the hospital main lab. Serum glucose, triglycerides, total and high-density lipoprotein (HDL) cholesterol, liver function tests and uric acid were measured using commercial methods (ADVIA® 2400 Chemistry System, Siemens Healthcare Diagnostic, and Deerfield, IL). Normal range for serum glucose was 60 to 100 mg/dl. Insulin was measured by a two-site sandwich immunoassay using direct chemiluminescent technology, requiring constant amounts of two antibodies (ADVIA® Centaur XP Immunoassay System; Siemens Healthcare Diagnostic, Deerfield, IL). The first antibody is a monoclonal mouse anti-insulin antibody labeled with acridinium ester. The second antibody, in the Solid Phase, is a monoclonal mouse anti-insulin antibody, covalently coupled to paramagnetic particles. The mean intra- and inter-assay coefficients of variations were 3% and 6%. Normal range for fasting insulin was 36–162 pmol/l.

Serum levels of DHEAS were measured by the routine laboratory immunometric methods on the Immulite 2000 autoanalyzer using commercial kits (Diagnostic Products Corporation (DPC), Los Angeles, CA, USA). DHEAS detection limit was 0.4 µmol/l, and CV was 7.1–7.2%. Serum levels of E_2_, testosterone, FSH and LH were measured by the routine laboratory immunometric methods on the Advia Centaur autoanalyzer using commercial kits (ADVIA® Centaur XP Immunoassay System; Siemens Healthcare Diagnostic, Deerfield, IL). β2 Estradiol (E_2_) detection limit was 0.05 nmol/l (functional sensitivity), and CV was 8.6–10.3%. Testosterone detection limit was 0.35 nmol/l, and CV was 8.2–9.9%. FSH detection limit was 0.3 IU/l, and CV was 4.2–4.5%. LH detection limit was 0.07 IU/l, and CV was 4.5%.

### Statistical analysis and data analysis

Continuous data are reported as median and range, with categorical data as counts and percentages. The Wilcoxon-test and the nonparametric Spearman correlation coefficient were used for intra-group comparison and correlation between continuous variables. Correlation coefficients for WBISI were age-adjusted. Simple and stepwise linear regression analyses were run to identify predictors of glucose concentration at 120 minutes (2HG), WBISI, ISSI-2 and BCDI at the follow-up visit. Models were adjusted for sex, age, BMI and duration of the follow-up. The p value was set as statistically significant at p<0.05. Data analysis was performed using SPSS statistical software (SPSS V15.0, Inc., Chicago, IL).

## Results

**Table 1** shows anthropometrics and metabolic parameters of patients at baseline and follow-up. The 47 obese children were re-evaluated after a median follow-up of 2.23 (1–4.52) y. Statistically significant differences were found in anthropometrics and values of metabolic parameters except for values of SBP, BMI-z score, IGI, BCDI and ISSI-2.

**Table 1:** Anthropometrics, laboratory and insulin metabolism-related parameters in preschool and school age patients.

	Obese cohort		
	Baseline (N = 47)	follow-up (N = 47)	*p*
**Sex (M/F)**	25/22 (53.2/46.8%)		-
**Age (years)**	5.16 (2.02–5.96)	7.19 (6.08–7.94)	<0.0001
**BMI-z score (SDS)**	3.42 (1.63–8.88)	4.77 (1.87–8.97)	0.9
**BMI (kg/m^2^)**	26.3 (17.9–35.5)	30.01 (20.5–38.7)	<0.0001
**Body weight (kg)**	34.5 (18.3–57.4)	52.3 (33.1–74)	<0.0001
**Waist circumference (cm)**	76 (62–95)	84 (74–102)	<0.0001
**Waist circumference (percentile)**	110 (91–140)	112 (72–132)	0.9
**Systolic blood pressure (mm/hg)**	106 (84–129)	108 (80–144)	0.2
**Diastolic blood pressure (mm/hg)**	60 (49–67)	63 (45–98)	0.02
**Fasting glucose (mmol/l)**	4.0 (2.94–5.05)	4.27 (3.05–5.32)	0.005
**Fasting Insulin (pmol/l)**	55.2 (13.2–336)	99 (36–460.2)	0.002
**2 Hour Glucose (mmol/l)**	5.36 (3.05–7.77)	6.24 (4.22–9.10)	0.001
**Total cholesterol (mmol/l)**	0.39 (0.16–0.58)	0.40 (0.29–0.62)	0.1
**HDL-cholesterol (mmol/l)**	12.3(6.72–18.3)	11.64 (7.75–18.6)	0.7
**Triglycerides (mmol/l)**	0.83 (0.26–2.52)	0.97 (0.41–1.97)	0.3
**HOMA-IR**	1.5 (0.35–8.54)	2.9 (1.1–12.12)	0.001
**WBISI**	5.88 (0.99–12)	3.34 (0.74–10.8)	0.0001
**AUC_G_ (mmol/l/min)**	5.31 (0.59–7.42)	5.92 (1.15–8.06)	0.8
**AUC_I_(pmol/l/min)**	475 (130.8–1171)	556.2 (99.6–2176)	0.9
**IGI**	0.95 (0.06–6.47)	2.20 (0.11–9.08)	0.07
**ISSI-2**	2.41 (0.95–4.95)	2.30 (1.19–5.89)	0.7
**BCDI**	0.18 (0.01–6.52)	0.69 (0.01–7.39)	0.07

Data are shown as median and range or number and % of individuals. *P* refers to statistical significance at the Wilcoxon test. β-cell demand index, BCDI; Body Mass Index, BMI; Area under the curve, AUC; Homeostasis Model Assessment of Insulin Resistance, HOMA-IR; Insulino-Genic Index, IGI; Insulin Secretion-Sensitivity Index-2, ISSI-2; Whole Body Insulin Sensitivity Index, WBISI.

Two patients in preschool age presented with concentrations of fasting glucose below the lower limit of normal values [i.e. FG = 2.95 mmol/l and 3.0 mmol/l, respectively], but values of fasting insulin were in the normal range (22.8 pmol/l and 49.2 pmol/l), respectively. One school age patient presented with an extremely high value of fasting insulin which peaked to 308 µUI/ml (1,848 pmol/l) following glucose load and did not return to the baseline value at hour 2. One child presented with values for glucose at 2 hours as high as 7.8 mmol/l at the baseline. IGT persisted in this child and overall four children (8.5%) were diagnosed with IGT at follow-up.

As regards pubertal development, at follow-up most children remained pre-pubertal (Tanner stage I), but four girls and six boys were classified as presenting early puberty (stage 2 for genitalia in boys or breast in girls and pubic hair stage 1). Eight of them underwent blood test for the assay of LH [0.03 (0.04–0.5) IU/l], FSH [1 (0.1–2.9) IU/l], E_2_ [82 (60–102) pmol/l], Testosterone [30.2 (22.1–90) pmol/l], and DEHAs [1,320 (980–1,980) nmol/l]. No statistical difference was observed between pre-pubertal and early pubertal cases in anthropometrics and metabolic profile including WBISI.

As regards gender differences, statistically significant differences were found at both baseline and follow-up. At preschool age, girls showed higher values of fasting insulin than boys [82.2 (22.8–336) vs. 44.4 (13.2–209.4) pmol/l, respectively; p = 0.007]. At school-age, girls presented higher values than boys of 2HG [6.88 (4.22–9.21) vs. 5.41 (3.49–6.88) pmol/l; p = 0.001], total cholesterol [0.42 (0.33–0.62) vs. 0.38 (0.29–0.52) mmol/l; (p = 0.04)]; and uric acid [309,2 (178.4–410.4) vs. 237.9(160.6–356.9) µmol/l; p = 0.02]. The change of ISSI-2 over the follow-up period was significantly greater (p = 0.02) in females (−97.99; −98.81 to −96.09) than in male patients (−97.30; −98.73 to −94.11).

### Correlations and regression models

Significant intra-individual correlations between values at baseline and follow-up were found in BMI z-score (*r_o_* = 0.745; p<0.0001), body weight (*r_o_* = 0.434; p = 0.002), BMI (*r_o_* = 0.410; p = 0.004), and waist circumference (*r_o_* = 0.395; p = 0.03), while no correlation was observed in indexes of insulin metabolism.

**Figure 1:**
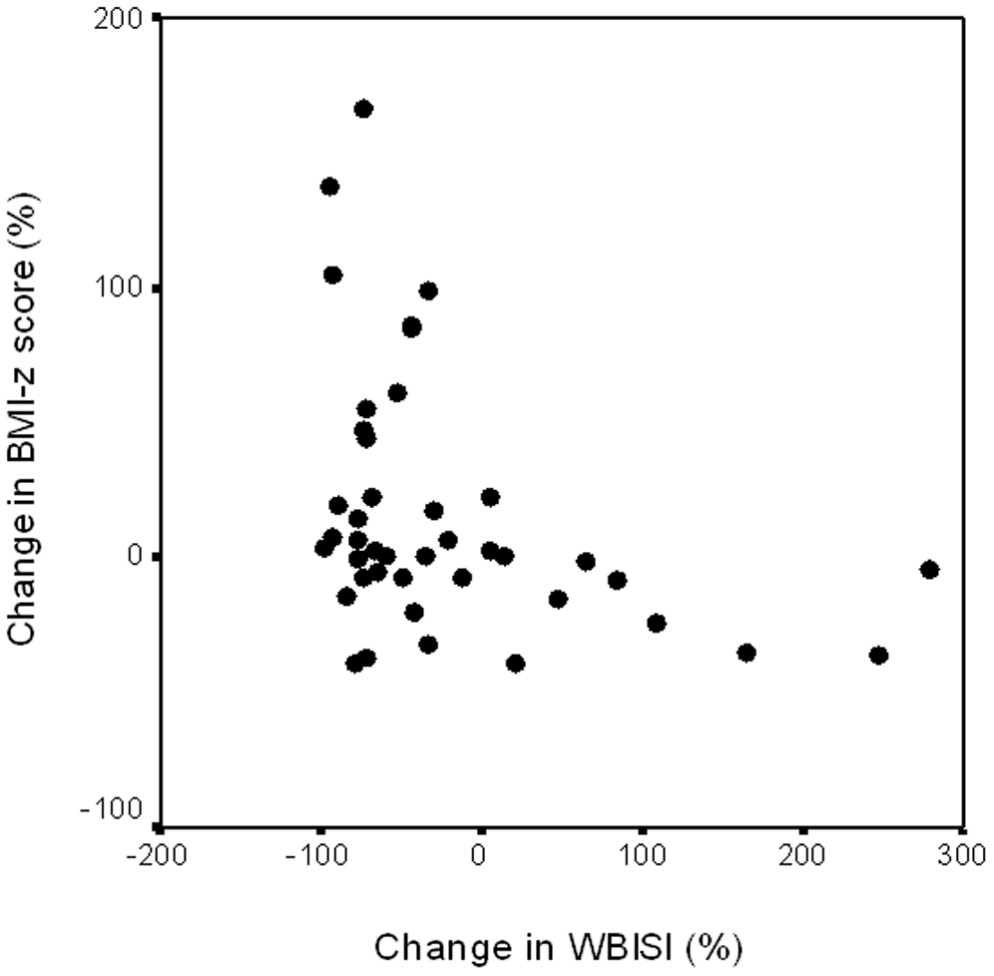
Relationship between percent changes in Whole Body Insulin Sensitivity Index (WBISI) and in BMI-z score (y = −0.6622x–11.194).

**Figure 2:**
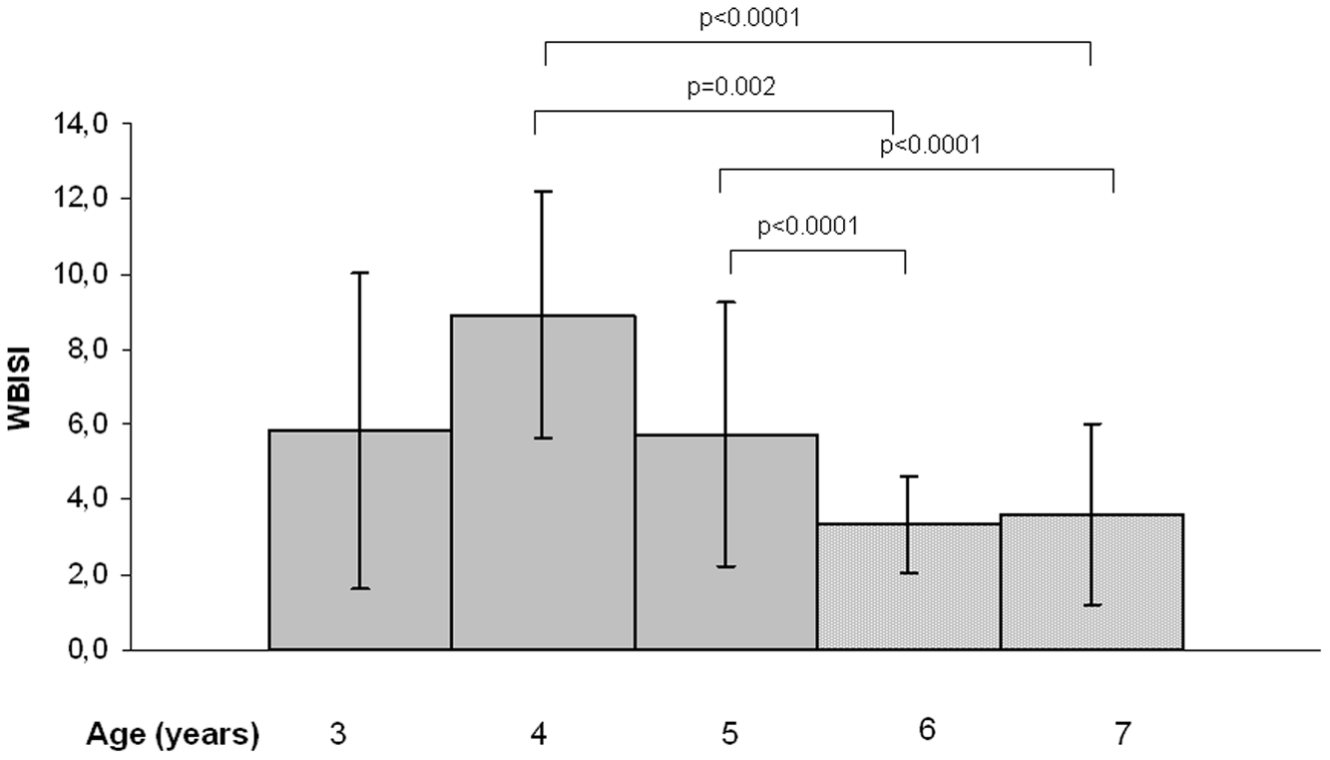
Mean values of Whole Body Insulin sensitivity (WBISI) by years of age in preschoolers and school age obese children.

**Table 2** reports *r_o_* values from Spearman correlation analysis for age-adjusted WBISI in preschool and school age obese patients. Changes of BMI-z score correlated significantly with changes of WBISI (*r_o_* = −0.400; p = 0.009); IGI (*r_o_* = 0.379; p = 0.013); 2HG (*r_o_* = 0.396; p = 0.01). **Figure 1** shows the association between changes in both WBISI and BMI-z score. Changes in WBISI were also correlated with age progression (*r_o_* = −0.324; p = 0.04). Indeed, **Figure 2** shows mean values of WBISI at different ages.

**Table 2:** Correlation analysis for age-adjusted WBISI in preschool and school age obese patients.

	Body-weight (kg)	BMI z-score (SDS)	Waist circumference (cm)	Waist circumference (centiles)
**Preschoolers Patients**				
**WBISI**	***r _o_*** ** = −0.604**	***r_o_*** ** = −0.420**	***r_o_*** ** = −0.545**	***r_o_*** ** = −0.479**
	***p*** **<0.0001**	***p*** ** = 0.005**	***p*** ** = 0.002**	***p*** ** = 0.006**
**Schoolagepatients**				
**WBISI**	*r_o_* = −0.087	***r_o_*** ** = −0.344**	*r_o_* = −0.241	*r_o_* = −0.209
	*p* = 0.6	***p*** ** = 0.02**	*p* = 0.1	*p* = 0.2

Linear regression models were run to better understand the relationship among changes in BMI z-score, waist circumference or lipid profile and insulin metabolism at follow-up. Variables that were statistically significant associated and those resulting with a p value<0.20 were successively modelled all together in stepwise regressions. Pubertal stage was put in all the stepwise models. WBISI at follow-up was predicted by changes in BMI z-score (R^2^ = 0.499; p = 0.034; β = −0.314); waist circumference (R^2^ = 0.168; p = 0.027; β = −0.410) and percentiles of waist circumference (R^2^ = 0.08; p = 0.027; β = 0.335). At the stepwise model, changes in BMI z-score predicted still significantly WBISI at follow-up(R^2^ = 0.309; p = 0.002; β = −0.556). BCDI at follow-up was predicted by changes in BMI z-score (R^2^ = 0.141; p = 0.010; β = 0.376); and circulating triglycerides (R^2^ = 0.068; p = 0.173; β = −0.260). Change in BMI z-score was the best predictor of BCDI (R^2^ = 0.246; p = 0.008; β = 0.496). ISSI-2 was predicted by changes in percentile of waist circumference (R^2^ = 0.071; p = 0.163; β = −0.266).

**Figure 3:**
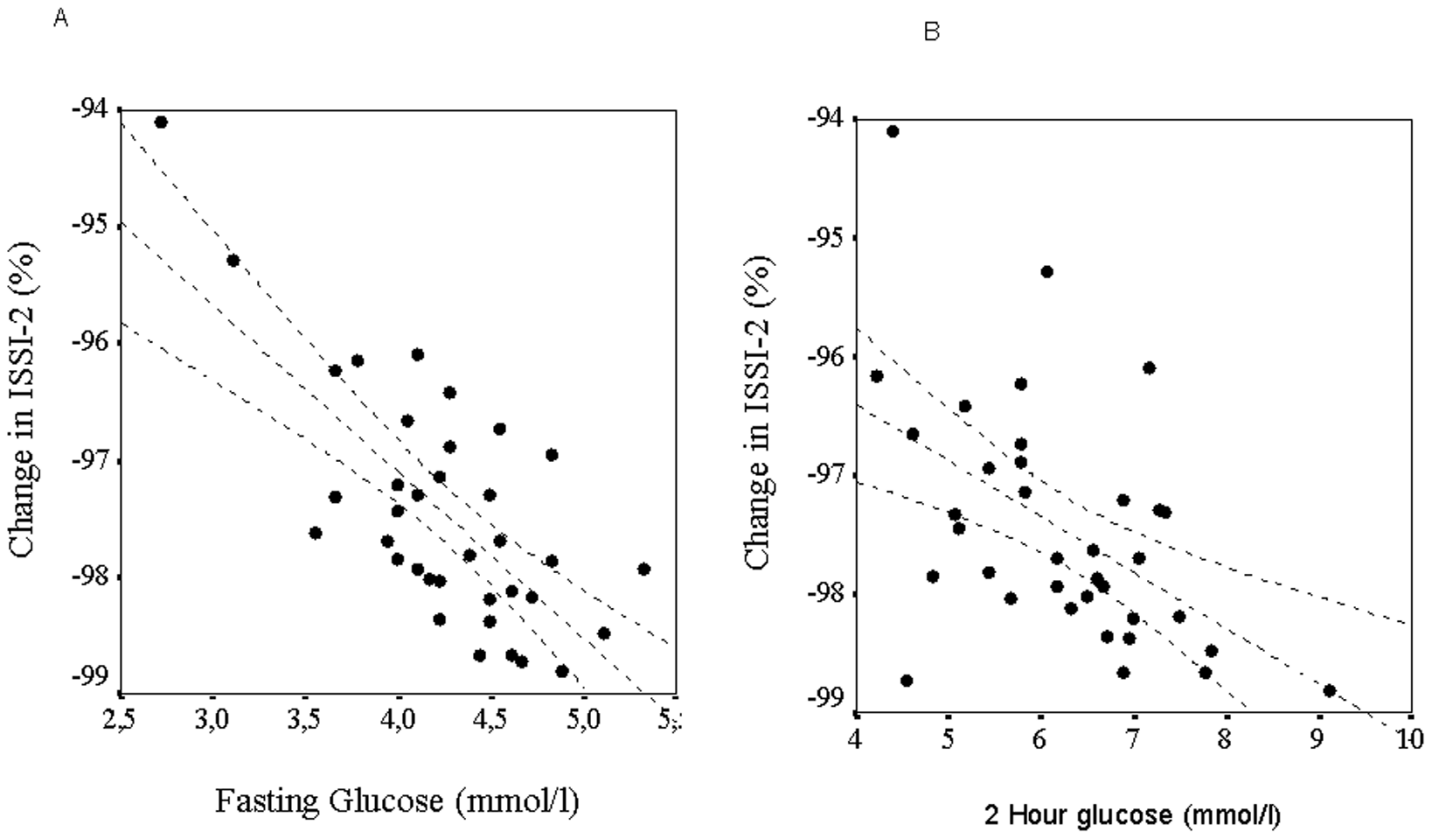
Relationship between percent changes over follow-up in the Insulin Secretion-Sensitivity Index 2 (ISSI-2), the oral glucose disposition index, and serum concentrations of fasting (Panel A; y = −5.8962x–497.65; p<0.0001; R^2^ = 0.492) and 2 hour serum glucose (Panel B; y = −11.207x–979.11; p<0.0001; R^2^ = 0.294) in school-age children. The decrease of the glucose disposition index over the follow-up period was associated with higher values of fasting and 2 h glucose in school-age children.

2HG at follow-up was predicted by changes in WBISI (R^2^ = 0.103; p = 0.024; β = −0.329); disposition index as estimated by the ISSI-2 (Panel B; R^2^ = 0.294; p<0.0001); IGI (R^2^ = 0.054; p = 0.138; β = −0.233); BMI z-score (R^2^ = 0.035; p = 0.210; β = 0.186). **Figure 3** shows the relationship between changes in ISSI-2 over follow-up and fasting glucose (Panel A; R^2^ = 0.492, p<0.0001) and 2HG in school-age children.

## Discussion

This is the first report on insulin sensitivity and β-cell function in preschoolers affected by severe obesity and on longitudinal changes occurring in insulin metabolism at transition from preschool to school age estimated by two serial OGTTs. Insulin sensitivity as estimated by the WBISI declined by almost 21% over 2 y of follow-up. Some but not all of the decline in insulin sensitivity could be explained by changes of the BMI z-score.

Our findings partly confirm results from the Early Bird Diabetes Study [12], a prospective cohort study of healthy children aged 5–14 years, which found that insulin resistance as estimated by the HOMA-IR rose progressively from age 7, three-four years before early puberty (Tanner stage 2). In our series, insulin sensitivity starts declining by age 5 years (Figure 2). The higher BMI of children in our series respect to normalweight children in the Early Bird cohort may explain some of the discrepancy in results. In the Early Bird, adiposity estimated as BMI-z score explained a small percent of the variation in insulin sensitivity (12% in boys and 20% in girls versus ∼30% in our series). In our series, the insulinogenic-index tended to increase in parallel with the statistically significant decrease of insulin sensitivity, allowing to maintain the glucose disposition index unchanged and to compensate for the increased β-cell demand index. Indeed, fasting and 2 h glucose at the school-age were predicted by the change in the glucose disposition index. Four children in our series presented impaired glucose tolerance by the age of 8 y. In particular, one of them had borderline 2 hour glucose value when he was in the preschool age.

Findings from the present study partly confirmed, in the sample of obese children, the metabolic paradox pointed out by the Early Bird Study [26]. Median insulin resistance was higher in school age than in preschool cases, hence supporting the concept that the decrease of insulin sensitivity begins before pubertal transition. Nevertheless, BMI z-score is only one of the factors influencing the prepubertal rise in insulin resistance and, importantly, deterioration of insulin sensitivity at this age is not accompanied by worsening of the lipid profile.

The strength of the present study is the longitudinal observation of insulin metabolism-related parameters since preschool age in severely obese infants. To the best of our knowledge, no past study has endowed with longitudinal information on insulin dynamics in obese preschoolers. However, despite the great novelty of the information provided, we are aware of the several shortcomings/weaknesses of our investigation. Ethical concerns prevented us from investigating WBISI values in age matched normal-weight controls and, hence, the study lacks controls. A stronger design would have been a prospective study, with controls consented to undergo OGTTs. We adopted OGTT derived indexes of insulin action and release which have been validated in children, but not as young as 4 years old [27]. Given the retrospective nature of the present investigation, we cannot provide information on the genetic background of obese patients, their family history of type 2 diabetes and body composition as estimated by more reliable techniques. Indeed, the retrospective design may have prevented adjustment for relevant covariates (i.e. family history of diabetes) in the adjusted analyses.

Ten children presented with early puberty at the follow-up. However, this latter finding does not undermine the idea that deterioration of insulin sensitivity begins early before puberty since whole body insulin sensitivity was not different between prepubertal and pubertal cases. The surge of LH in these patients was not different from that observed by Jeffery et al in the Early Bird study [12]. Nevertheless, we are aware that the fact that there were no significant differences between pre-pubertal cases and the early pubertal cases may be due to limited power in the very small sample.

Quite modest sample size, high age variability in the preschool age and variable length of follow-up represent major drawbacks. Finally, we are not able to rule out if the changes observed in the 47 obese children are specific to the obese cohort or reflect the changes that one would observe in the general population.

In conclusion, insulin sensitivity in morbidly obese children seems to decline even before pubertal transition. Changes in total adiposity partially explain the variation of insulin sensitivity, but further studies are warranted to identify other determinants of such decline.

The deterioration of the glucose disposition index seems to determine the glucose tolerance later in the school age. More importantly, in highly morbidly obese children overt impaired glucose tolerance may occur unexpectedly early.
